# Use of Polycaprolactone Electrospun Nanofiber Mesh in a Face Mask

**DOI:** 10.3390/ma14154272

**Published:** 2021-07-30

**Authors:** Morshed Khandaker, Helga Progri, Dhakshyane Tamil Arasu, Sadegh Nikfarjam, Nabila Shamim

**Affiliations:** 1 Department of Engineering and Physics, University of Central Oklahoma, Edmond, OK 73034, USA; hprogri@uco.edu; 2 Department of Biology, University of Central Oklahoma, Edmond, OK 73034, USA; dtamilarasu@uco.edu (D.T.A.); snikfarjam@uco.edu (S.N.); 3 Department of Chemical Engineering, Prairie View A&M University, Prairie View, TX 77446, USA; nashamim@PVAMU.EDU

**Keywords:** electrospun, nanofiber, polycaprolactone, microbial, filter

## Abstract

Electrospun nanofiber mesh has previously been used as an air filtration device. However, the qualification of polycaprolactone (PCL) nanofiber mesh cloth in face masks to protect individuals against airborne particles carrying microorganisms has yet to be investigated. The long-term goal of this study is to develop methods to use PCL nanofiber mesh to provide better protection against microorganisms. To achieve this goal, we observed the morphology, water droplet absorption, thermal (differential scanning calorimetry), mechanical, and airborne particle filtering capabilities, and also the microbial activities of a PCL cloth, to evaluate whether it is suitable to act as a filter in a face mask. We have produced a polycaprolactone (PCL) nanofiber cloth after electrospinning it onto a drum for 3 and 10 min, referred to hereafter as PCL-3 and PCL-10, respectively. Our study found that the middle protection layer (control) of the Henry Schein Earloop Procedure Mask contains pores (average diameter = 5.72 ± 0.62 µm) which are 48 times larger than the diameter of a microorganism an average diameter of ~120 nanometers. However, PCL-10 nanofiber membranes show pores with an average diameter of 1.42 ± 0.34 µm. Our contact angle measurement tests found that all the samples were very hydrophobic (contact angle values varied between 120 and 150 degrees). However, both PCL cloths’ contact angle values were lower compared to the control. The produced PCL cloths showed a lower water droplet absorption compared to the control. Thermal studies found that PCL is stable in extreme conditions and no plasticizing effect occurs due to the presence of a solvent. Mechanical tests showed that PCL-10 cloth had higher strength and modulus compared to the control and PCL-3 under tension loading conditions. A vacuum experiment found that the PCL-10 fiber cloth could withstand a negative pressure of 18 Psi without any signs of breakage, and the mask was able to capture airborne particles and microorganisms. The feasibility of immobilizing anti-bacterial nanoparticles with PCL during electrospinning creates the future potential of producing an anti-bacterial face mask using PCL.

## 1. Introduction

Filters are used widely in our daily lives, from kitchen sinks to machinery. However, certain requirements need to be met when trying to classify a material as a potential filter. Due to the recent outbreak of the coronavirus, this study is motivated by the process of constructing a filter made out of bioengineered cloth to protect individuals against the virus. There are four main properties of a filter; firstly, they are required to be durable, flexible, and sturdy. A material like this will not be affected by the maximal human expiratory (233 ± 84 cm H_2_O) and inspiratory (66 ± 32 cm H_2_O) pressures [1]. The second aspect of a filter is the resistance to water droplets [2]. A proper filter should exhibit hydrophobic properties since this virus is known to spread through respiratory droplets [3]. In addition to hydrophobicity, filters need to be designed to prevent airborne particles from passing through it [4]. This is related to the diameter of the pores present in the filter and how densely the bioengineered cloth is manufactured. Finally, filters should have antiviral or anti-bacterial properties in order to eliminate any form of pathogen attachment and provide proper protection [5].

In the current state of affairs, medical professionals now have to rely on normal procedure masks, which do not protect individuals against the coronavirus, as our studies show that the size of the pores is much bigger than the diameter of the virus. This poses a threat to many individuals. Many pieces of protective gear are being rejected as they do not provide sufficient filtering properties, such as 3D printed masks and surgical masks. The only mask in the market that currently provides sufficient protection from microorganism-related diseases is the N95 mask [6]. This is because N95 masks prevent the user from inhaling small airborne particles in aerosol-generating procedures; also, it fits tightly to the user’s face [7]. This mask must be worn with a form of eye protection in addition to a standard surgical mask. There are three categories for N95 masks: FFP1, FFP2, and FFP3 [8]; FFP3 has the highest level of protection. There are three protection layers in the N95 mask, which can filter airborne droplets of up to 0.3 micrometers (µm), whereas there is only a single layer of protection in a general-purpose surgical or procedural mask. These general-purpose surgical masks only filter airborne particles of up to 425 microns or +0.452 µm [9]. A recent study found that airborne droplets with sizes varying from 0.05 to 500 μm [10] consist of sub-micron droplets directly emitted due to respiratory activities, and the droplet nuclei formed from the evaporation of super-micron droplets contain viruses of size 0.02–0.3 μm [11]. Therefore, a general-purpose surgical or procedural mask is unable to protect humans from droplets with particles or organisms of size less than 0.452 µm.

PCL nanofiber matrix is widely used in tissue engineering applications [12]. However, the usage of PCL nanofibers in terms of the effect of electrospinning time on porosity, contact angle, water absorption, thermal stability, mechanical properties, filtration capabilities, and microbial properties has not been ventured into. PCL is a biocompatible and FDA-approved material [13]. Depending on the production, PCL nanofiber cloths can be produced with a much smaller pore diameter to serve as a filtering tool. PCL cloths can also protect an individual from airborne particles containing microbial agents from entering the respiratory airways, as anti-bacterial agents can be immobilized onto the PCL cloth [14]. According to Zhang Z et al. [15], most viral transmissions occur via horizontal transmission, and viruses pass through human-to-human contact. The authors reported the use of electrospun ultrafine fibers for advanced face masks. Several other pieces of research reported COVID-19 virus transmission can be prevented by introducing protective electrospun nanofiber membrane barriers or filters in face masks [16]. In addition to a protective barrier in masks, this filter may also assist different industries or fields that require its products to be filtered prior to them entering the environment. We have conducted a series of test in this study showing that PCL nanofiber cloths can be a potential candidate for protection against the coronavirus, as the characteristics of PCL cloths provide great features such as filtering, hydrophobicity, and flexibility. No studies are currently being conducted with respect to PCL nanofibers acting as a protective layer in masks. Due to the advantage of incorporating antimicrobial nanoparticles into PCL to target a specific microorganism, the use of PCL in face masks is versatile and not limited to protecting against the COVID-19 virus. The ultimate goal of this study is to develop methods for providing better protection against the novel coronavirus.

## 2. Materials and Methods

### 2.1. Materials

This study used poly (ε-caprolactone) beads (pellet size ~3 mm, average M_n_ 80,000) and acetone (laboratory reagent ≥99.5%) to prepare the PCL nanofiber cloth. Both the PCL and acetone were purchased from Sigma Aldrich (Sigma-Aldrich Co. LLC., St. Louis, MO, USA). We purchased Henry Schein Earloop surgical masks (ASTM F2100 Level 3 Surgical Mask) to prepare the control samples. The middle layer of the surgical mask was used as a control sample, and the electrospun PCL cloths of varying thickness were used as test samples. Typically, the middle filtration layer of a surgical mask is covered internally and externally by polypropylene fabric. We bought polypropylene fabric from Walmart to prepare samples for the filtration and microbial analysis. 

### 2.2. Sample Preparation

**Figure 1:**
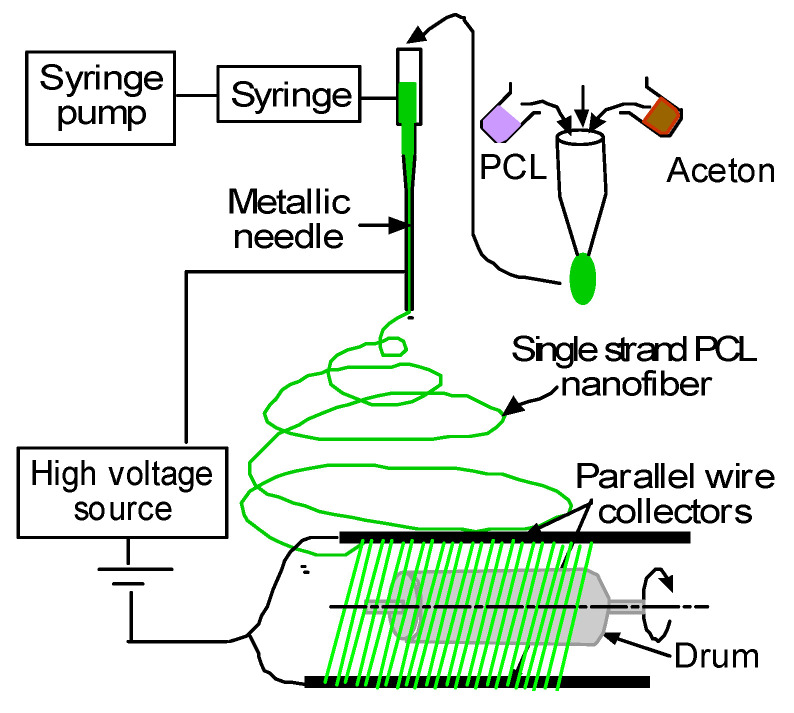
Schematic representation of an electrospun nanofiber production system that can be set up to produce PCL.

PCL/acetone solution was prepared as described in our previous research [17]. The concentration of PCL in the solution was 10 wt.%. Figure 1 shows an image of the nanofiber fabrication process that was followed to produce PCL nanofiber cloths with a specific thickness. A syringe pump was used to feed the PCL solution into a glass syringe and flow it through a tube with a metallic needle. The drum collector was spun using a direct current (DC) motor. The syringe needle was electrically excited by applying high voltage (9 kV) produced by the Gamma High Voltage power supply. This electrically charged syringe needle was positioned above a drum collector to capture the PCL-aligned fiber stream. The distance between the needle and drum collectors was approximately 5 cm. The feeding rate of the PCL solution was adjusted to a rate of 0.025 mL/minute. PCL cloths were directly collected on a drum with a 2-inch diameter. We optimized the speed of rotation, the distance between needle and drum, and the deposition rate of fiber onto the drum. We produced an evenly coated nanofiber cloth of two different thicknesses used for all our studies by electrospinning them for 3 and 10 min, referred to in this study as PCL-3 min and PCL-10 min cloths. A sterilized sharp razor blade was used to cut the PCL cloth into dimensions of 18 cm long and 16 cm wide from the drum to be used as different study (SEM, water absorption, thermal, mechanical, filtration, and microbial) samples. Samples were stored in an autoclave bag.

### 2.3. Scanning Electron Microscope and Morphology

**Figure 2:**
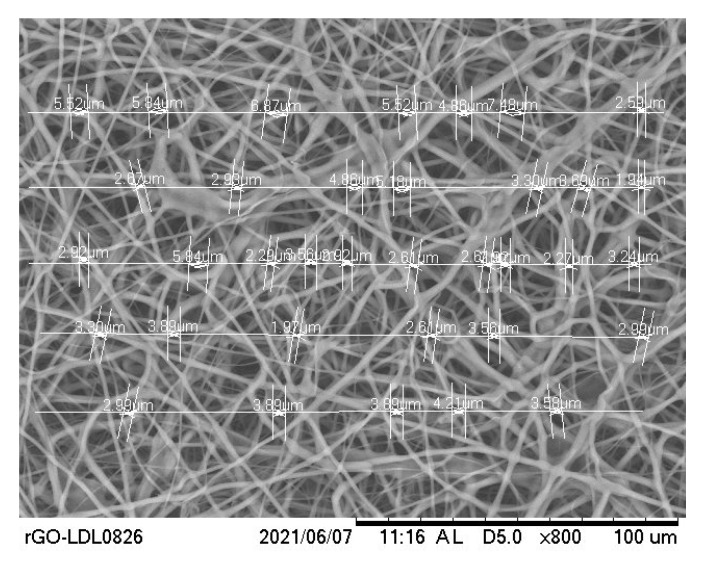
Method of pore size measurements using line intercept method from a Scanning Electron Microscope (SEM) image using ImageJ software.

A 5 mm biopsy punch was used to cut off 5 mm diameter control, PCL-3, and PCL-10 samples from the produced cloth. The thickness of the produced fiber was measured using a digital caliper (±0.02 mm). The topography of fibers on carbon tape was visualized using the Zeiss Neon 40 EsB (5 kV with Inlens or SE2 detector specified on the image) (Wetzlar, Germany) and the Quattro ESEM (Thermofisher Scientific, Waltham, MA, USA). For the Zeiss Neon, the samples were sputter coated with approximately 5–6 nm of AuPd to visualize the pore size correctly. For the Thermofisher Quattro S, the samples were not coated. We used the low vacuum mode at 10 kV and the CBS detector. The pore size of each sample was analyzed using scanning electron microscope software (Hitachi TM 3000) (Tokyo, Japan). SEM analysis was conducted to measure the pore size of the control, PCL-3, and PCL-10 cloths using a line-intercept method which is commonly used to measure grain size diameters [18]. From a captured SEM image (Figure 2), five arbitrary lines were used to measure the average gap size (or pore diameter). ImageJ processing software (National Institute of Health, Bethesda, MD, USA) was used to measure the average pore diameter, which was considered to be the gap size between two fibers. The measurement tools in ImageJ software were used to measure the sizes of the gaps on those lines. The mean pore diameter was reported. The SEM image was also used to evaluate whether the cloth was capable of capturing airborne particles. All images were captured at 800× magnification. 

### 2.4. Contact Angle 

A contact angle goniometer (OCA 15, Future Digital Scientific Corp., Westbury, NY, USA) coupled to a high-definition and high-speed digital camera (1 min, 25 frames/s at 25 C) was used to measure the contact angle of a droplet on the control, PCL-3, and PCL-10 cloths. The tests were conducted at the Department of Restorative Sciences, The University of Oklahoma Health Sciences Center College of Dentistry. Contact angle results were analyzed using the Laplace-Young equation. A detailed description of the contact angle measurement method which was followed in the study is given in Garner et al. [19]

### 2.5. Water Absorption

**Figure 3:**
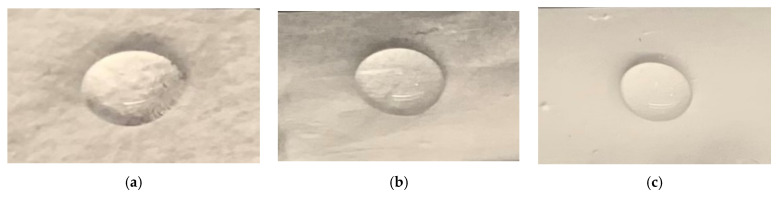
The architecture of 100 µL water droplet after 5 min on (**a**) control, (**b**) 3 min electrospun, and (**c**) 10 min electrospun cloth used during water absorption analysis.

A piece of cloth with dimensions 2.5 cm × 1.7 cm (length × width) was cut from the 15 cm × 20 cm piece for the water absorption analysis of the control, PCL-3, and PCL-10 samples (Figure 3). Each sample weight was measured using a precision scale (W_0_). A water droplet of volume 100 µL was placed on each sample (*n* = 3/sample group) for 5 min at room temperature using a pipette. The reason for 100 µL water droplets is that mucosaliva launched from a height of 1.5 m directly in front of the face is expected to have a volume of 86.6 μL, or a single droplet with a diameter of 14 μm, which is close to our lower limit droplet size range [20]. After 5 min, each sample was vacuum dried and the weight was measured (W_t_). The percentage of water absorption of each sample was measured using (W_t_ − W_0_) × 100%/W_0_ and compared.

### 2.6. Thermal Analysis

The thermal analysis of the electrospun nanofiber was carried out using a Mettler-Toledo DSC 3 (Mettler-Toledo Ltd., Port Melbourne, Australia) to study the effect of the cooling rate on melting temperature. Heating scans were performed from 25 to 120 °C after cooling at rates ranging from 0.3 to 20 K/min. The heating rate was fixed at 10 K/min. Calibration of the instrument was performed following the manufacturer’s procedures and using indium. Due to the light and fluffy nature of electrospun nanofibers, they were folded and cut into smaller pieces to put into the aluminum hermetic pan with a closed lid for experimental processes. The concentration of PCL was kept constant at 10 wt.%. Electrospun fibers typically contain residual solvents from the spinning process and must be dried before measurements. The samples were heated to 120 °C, and then the sample had a short isothermal hold for 1 min to reach equilibrium. The samples were then cooled at a specific cooling rate. Similar experimental procedures were followed for the cooling rates ranging from 0.3 to 20 K/min.

### 2.7. Mechanical Tests

**Figure 4:**
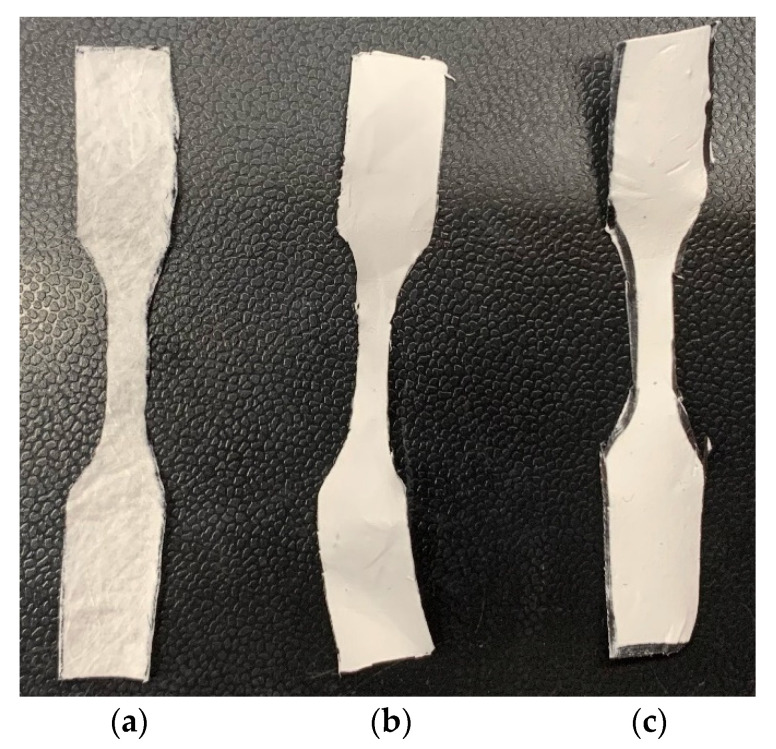
Tension test samples: (**a**) control, (**b**) PCL-3, and (**c**) PCL-10.

Five pieces of control, PCL-3, and PCL-10 cloth with dimensions 10 mm × 3 mm (length × width) were cut from the 15 cm × 20 cm piece using a razor blade (Figure 4). Control samples with the same dimensions were cut from the middle layer of Henry Schein Earloop surgical masks. Pull-out tension tests were conducted on each sample to measure elastic mechanical properties under tension. The tension tests were carried out using the CellScale Biomaterial mechanical test system (Waterloo, Ontario, Canada), which was programmed to provide a constant compression rate of 1 mm/min to each test sample. The stage was attached to a 300 N load cell and 60 mm stroke length displacement sensors to record force and displacement values from the tension tests and calculate corresponding stress and strain values. The test system was equipped with a camera to capture the displacement during the mechanical test every 30 s. The study reported the tension modulus and maximum tensile stress calculated from the calculated stress and strain values. The strain was calculated by dividing displacement over the grip length, and stress was calculated by dividing stress over the initial cross section area (width × thickness) at the middle of the sample.

### 2.8. Filtration Analysis

**Figure 5:**
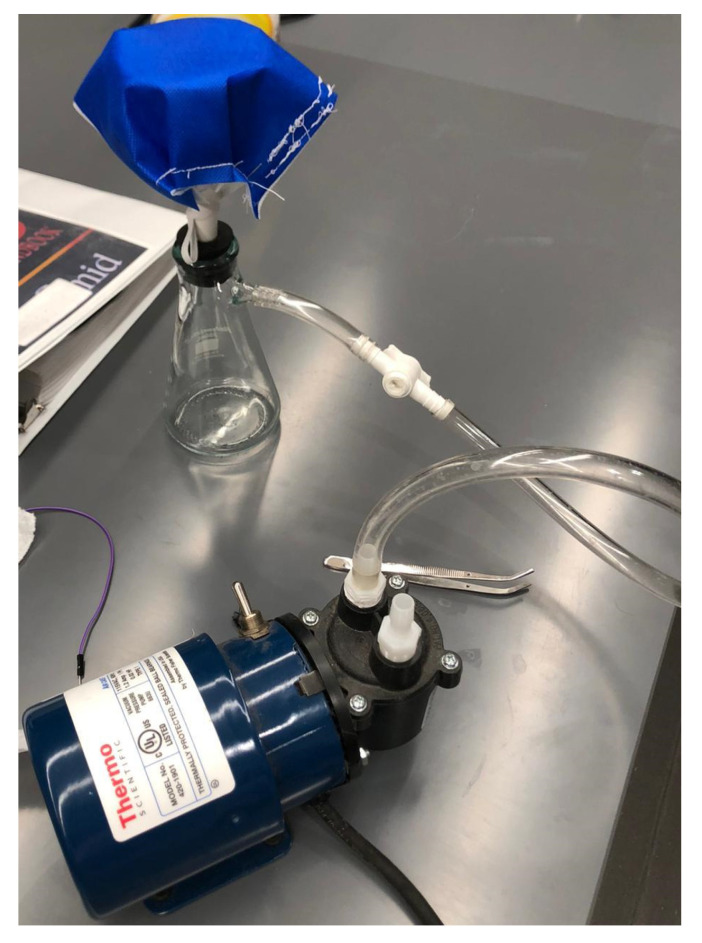
Measurement of airborne particle absorption capability.

This study produced a surgical mask made of 18 cm long and 16 cm wide PCL-3 and PCL-10 cloth. Each cloth was inserted between two pieces of polypropylene fabric with the same dimensions (18 × 16 cm) and sutured along the edge using SINGER 01664 Stitch Sew Quick 2 Hand Held Mending Machine. A vacuum pump (Thermo Scientific model #420-1901, air capacity 0.6 cfm), as shown in Figure 5, was used to measure the capability of capturing airborne particles by each cloth. Two types of samples were tested using this setup; the procedure mask, which serves as a control, and the fabricated mask containing PCL-3 and PCL-10 cloths. Each sample was left on the vacuum pump for approximately 10 min. The setup was placed in a laboratory at room temperature. The samples were then observed under an SEM microscope to determine the airborne particle absorption level.

### 2.9. Microbial Analysis

Upon University of Central Oklahoma Institutional review board (IRB) approval, six students volunteered to wear the PCL-10 masks and surgical control masks for a day. Three students wore the Henry Schein Earloop surgical mask (control) and three wore the PCL-10 nanofiber masks for a day. The mask was treated cautiously so that only airborne bacteria could come into contact with the middle layer of the mask. Students kept masks in a sterilized autoclave bag when they were not wearing the masks. The middle layer of Henry Schein Earloop surgical mask (control) and PCL-10 mask (test) were removed carefully by cutting the edges and stored in a sterilized Petri dish. Five separate areas of the masks were sliced before being inoculated with swab pods and performing the Gram staining procedure in the middle layer of Henry Schein Earloop surgical mask (control) and PCL-10 mask.

We also conducted an anti-bacterial test using the Kirby Bauer disc diffusion method. Three tryptic Soy Agar (TSA) plates were prepared and staphylococcus aureus was inoculated and spread throughout the plates. A biopsy punch cut out 8 mm diameter discs from the control, PCL-3, and PCL-10 cloths. Each group of sample discs was placed on the plate with the spread bacteria. The plate with the sample groups was incubated overnight and the inhibition zone diameter was examined.

## 3. Results

### 3.1. Fabricated Samples

**Figure 6:**
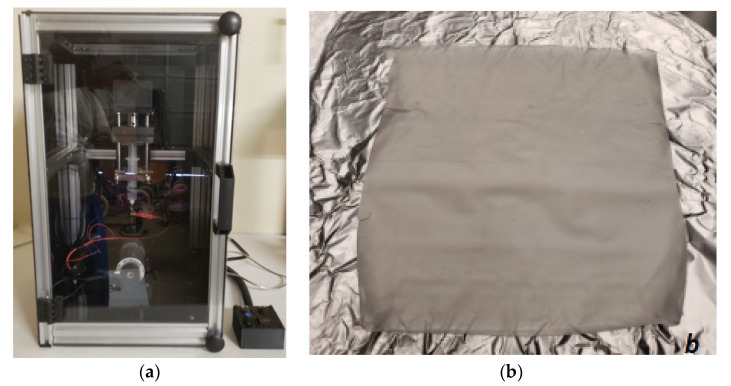
(**a**) Fiber collection on a drum using an electrospinning unit and (**b**) a produced PCL cloth after 10 min of electrospinning from the drum.

This study successfully produced PCL fiber cloths on a drum collector after spinning the drum for 3 and 10 min using an electrospun fiber machine (Figure 6a). Figure 6b shows a PCL cloth cut out from the drum using a razor blade. Although each cloth was produced under similar laboratory conditions and following the same flow rate and distance between needle and drum, the fiber density was not consistent for either of the PCL-3 and PCL-10 cloths. We were cautious when cutting samples for the purpose of SEM, water absorption, thermal, and mechanical tests from the drum.

### 3.2. Morphological Analysis

**Figure 7:**
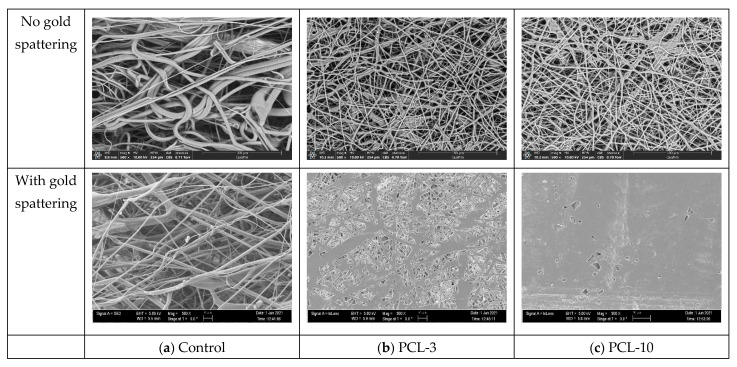
Side by side, SEM images of without and with gold spattering: (**a**) control, (**b**) PCL-3, and (**c**) PCL-10 cloths showing the difference of pores that exist in the samples. All images were taken at 500× magnification.

The average thicknesses of the control, PCL-3, and PCL-10 samples were measured to be 0.11 ± 0.02 mm, 0.03 ± 0.01 mm, and 0.05 ± 0.02 mm, respectively. The Scanning Electron Microscope images show the presence of pores in the control, PCL-3, and PCL-10 samples (Figure 7). We observed a similar morphological structure for the control (Figure 7 left) and PCL-3 (Figure 7 middle) cloths. However, SEM images of the PCL-10 cloth (Figure 7 right) showed that there were fewer pores in the PCL-10 cloth than the control and PCL-3 samples. The fiber diameters in the PCL-3 and PCL-10 cloths ranged from 300 to 800 nm, whereas the control cloth had a fiber diameter ranging from 300 nanometers to 8 micrometers.

ImageJ pore analysis results from the line intercept method (Table 1) revealed no statistical difference in average pore size among the samples. Our SEM images show that PCL-10 was still fibrous, but due to the production of multiple layers, the pores became significantly lesser. There were no visible pores when we assembled two layers of PCL nanofiber membranes.

### 3.3. Contact Angle

**Figure 8:**
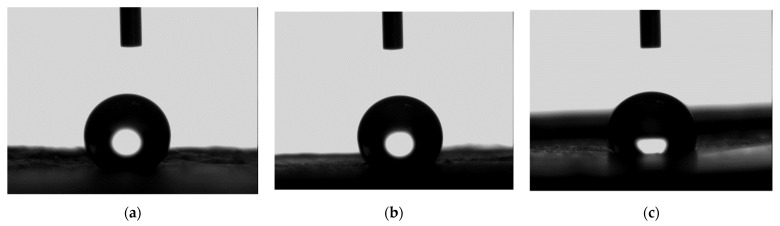
Representative images of a droplet from the contact angle measurement test: (**a**) control, (**b**) PCL-3, and (**c**) PCL-10.

Figure 8 shows representative images of a droplet during the contact angle measurement test. The initial average contact angles based on the first 10 test data for control, PCL-3, and PCL-10 were found to be 152.90°, 117.12°, and 136.81°, respectively. The contact angles after 60 s based on the last 10 test data for control, PCL-3, and PCL-10 were found to be 149.26°, 116.76°, and 138.46°, respectively. Our contact angle measurement test found that all the samples were very hydrophobic (contact angle values varied between 120 and 150 degrees). However, there was a difference in hydrophobicity between the samples. These results agree with other researchers’ findings [21].

### 3.4. Water Absorption

**Figure 9:**
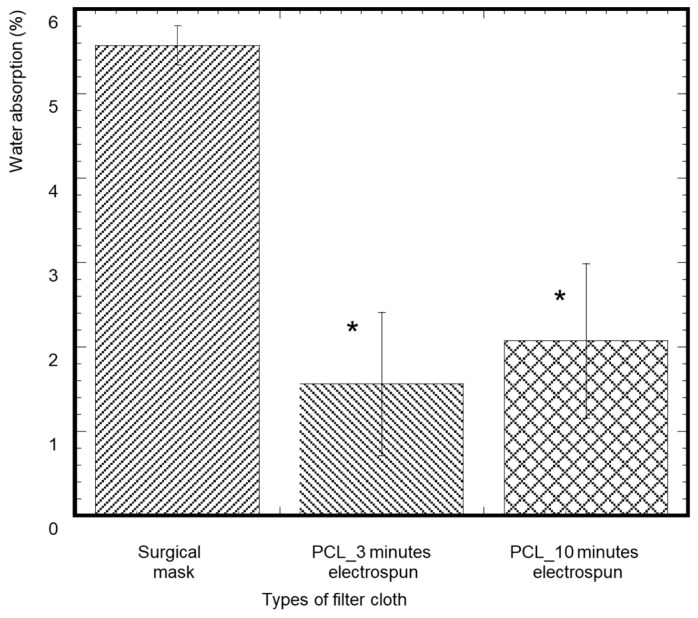
The percentage of weight difference of samples from the initial weight after applying water droplets for 5 min. The values of all parameters were reported as mean ± SOE (*n* = 3). * refers *p* < 0.05 with respect to control (surgical mask).

A significantly lesser amount of water absorption was observed in the PCL-3 and PCL-10 cloths compared to the control (Figure 9). This result is reasonable as the middle layer (control) in the surgical mask is usually made up of a hydrophobic material [22]. Both PCL cloths showed negligible water absorption since our study shows that both PCL cloths have hydrophobic properties. Although the value of weight increase was negligible, we found a higher percentage of weight increase for the PCL-10 samples compared to PCL-3. These results can be explained by the larger pore sizes in PCL-3 compared to PCL-10. The water droplets can filter out through those pores, which may result in less weight for PCL-3 compared to PCL-10. It is due to the dense fibrous layer of PCL-10 that the hydrophobic properties of PCL can be seen.

### 3.5. Thermal Test

**Figure 10:**
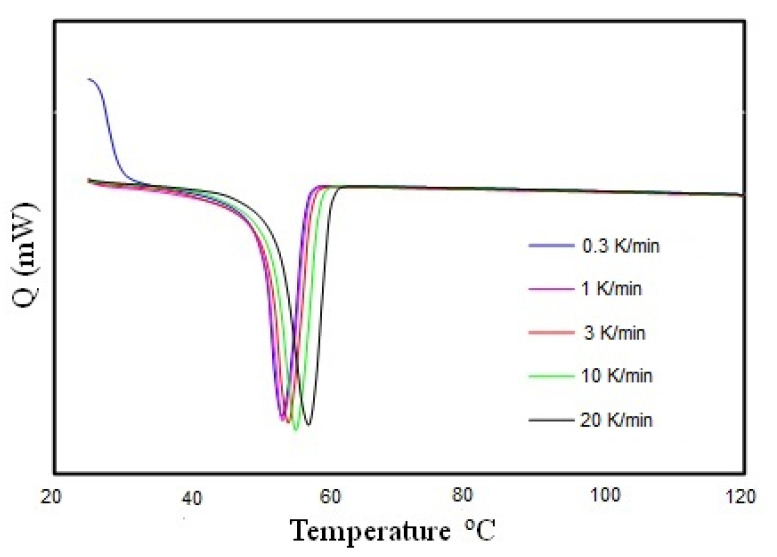
Heat flow curves of PCL nanofibers as a function of cooling rate q as indicated and heating rate 10 K/min.

**Figure 11:**
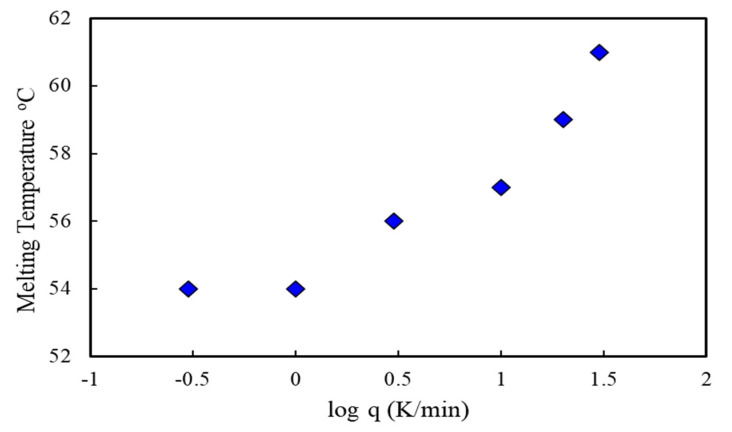
Cooling rate dependence on melting temperature for 10 wt.% PCL nanofibers.

PCL is a crystalline linear polyester, and its thermal properties, such as the degradability of PCL, depends on its molecular weight and degree of crystallinity. The degree of crystallinity can be measured using an X-ray diffractometer or from the integral melting heat determined by DSC. We studied the melting temperature of the PCL nanofiber by using a Mettler Toledo DSC 3 (Figure 10). Figure 10 shows the heat flow curves of PCL nanofibers as a function of cooling rate (q) ranging from 0.3 to 20 K/min with a constant heating rate of 10 K/min. Interestingly, the melting point (T_m_) shifted to a slightly higher temperature with an increasing cooling rate, as shown in Figure 11. The cooling rate depends on the melting temperature for 10 wt.% PCL nanofibers. This might be due to the crystallite size in the polymer nanofibers because at lower cooling rates or longer cooling times at any specific temperature, polymer chains get more time to arrange in crystalline order, which results in the variation of the melting temperature.

The effect of solvents on electrospun nanofibers was observed using DSC. The DSC heat flow curve shows a sharp melting point peak, which allows us to conclude that there is no plasticizing effect due to the remaining solvent. This confirms the fabrication of pure PCL nanofibers.

### 3.6. Mechanical Tests

**Figure 12:**
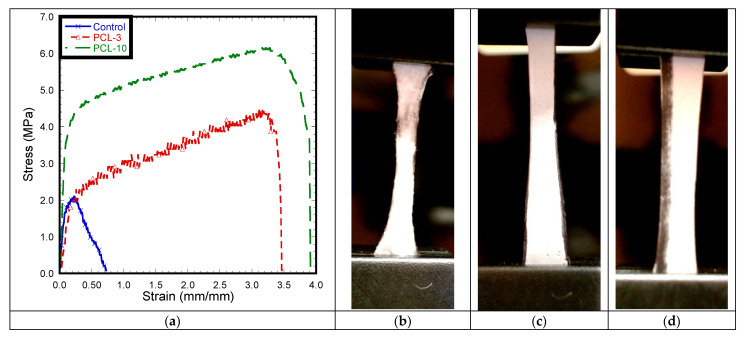
(**a**) Stress–strain curve of pull-out tension tests for (**b**) control, (**c**) PCL-3, and (**d**) PCL-10 cloths.

This study conducted mechanical tests on the control, PCL-3, and PCL-10 samples. During the tension test (Figure 12), there was a significant reduction of width observed for both PCL-3 and PCL-10 with no visible break of fiber from the edge, whereas breakage of fiber occurred on all control samples, specifically near the grip. As observed in the stress–strain diagram, the fluctuation of stress with applied strain was due to the breakage of control fiber inside the cloth. Pore size or void extension of PCL fiber cloth with increased load might be the reason for a long stretch at the higher load after yielding in the case of the tension experiment. Figure 12 shows a distinct yield point for each sample. A similar trend of stress–strain was observed for both the PCL-3 and PCL-10 samples. Both the PCL-3 and PCL-10 cloths showed a similar long stretching length after yielding until the breaking of the samples when a gradual decrease of load occured.

Table 2 reports that Young’s modulus and tensile strength for the PCL-3 and PCL-10 samples are significantly higher compared to the control. However, the results indicate no significant difference in Young’s modulus and tensile strength between PCL-3 and PCL-10. The results clearly show that a difference in material thickness due to different electrospinning times has an effect on the mechanical properties of PCL.

### 3.7. Filtration Analysis

**Figure 13:**
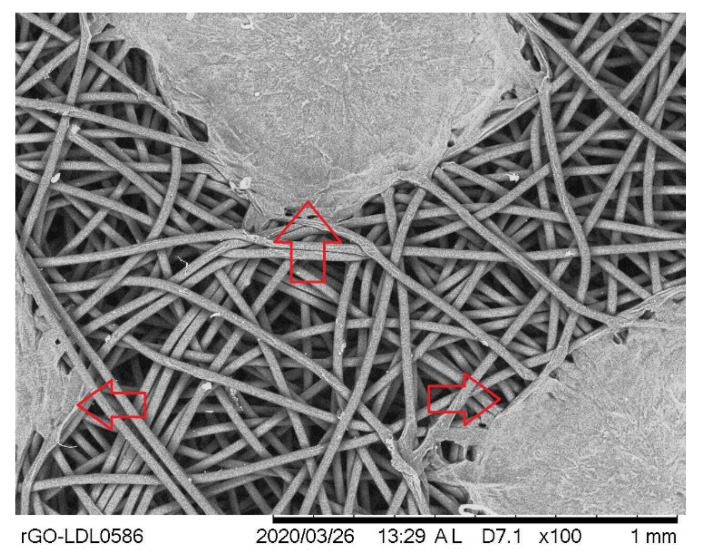
Airborne contaminant collection on PCL fiber cloth indicated with red arrows.

All samples were intact during the filtration test. This means that PCL cloth with an inner and outer layer made of protective cloth has sufficient stiffness to be used in a face mask. From the SEM image, we were unable to see any airborne particles in the control and PCL-3 cloths after the filtration test. However, the SEM image (Figure 13) revealed several clusters of airborne particles in the PCL-10 cloth after the test. The diameter of the captured particles at five arbitrarily selected places was measured to be 0.96 ± 0.10 mm. The filtration experiment was conducted with vacuum pump under maximum vacuum (20 inch Hg) and pressure (18 psi). The high vacuum pressure and large pore size in the control and PCL-3 samples might be the reason for the nonexistence of airborne particles in the cloths.

### 3.8. Microbial Analysis

**Figure 14:**
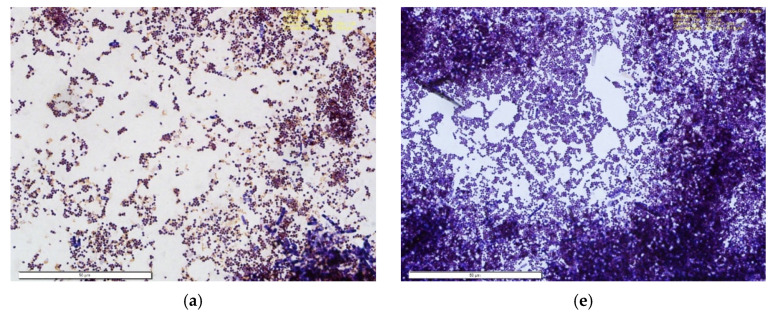

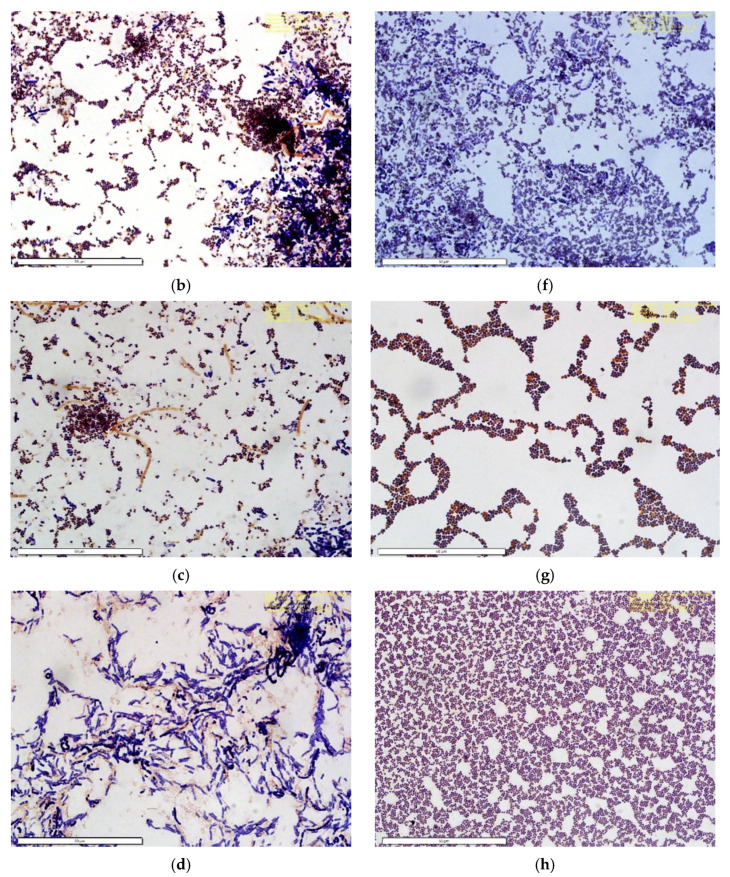
Illustration of Gram staining of two study groups—the control (**a**–**d**) (left) and PCL nanofiber mask (**e**–**h**) (right) used to compare the morphology and characteristics of bacteria in each group. (**a**,**b**) show low condensation of Gram-positive cocci and high accumulation of Gram-negative cocci, whereas (**e**,**f**) depict vast amounts of high Gram-positive cocci accumulation. (**c**) shows both Gram-positive and -negative cocci, bacillus, and spirochetes in lesser amounts. (**d**) shows a high amount of Gram-positive and -negative bacillus. (**g**) shows the presence of both Gram-positive and -negative cocci and (**h**) shows a high accumulation of Gram-negative cocci.

The Gram stain revealed the presence of both Gram-positive and Gram-negative bacteria: Coccus (sphere-shape) and Bacillus (rod-shape) bacteria that stained dark purple and red or pink due to the retention of the primary dye (Crystal Violet) or the counterstaining dye (Safranin) in the cell wall of the bacteria embedded on the nanofiber mask [23]. According to the analysis, out of the 15 cuts for each test group (30 samples total) of 1 cm × 1 cm, 42% were Gram-negative cocci, 53% Gram-positive cocci, 3% Gram-positive bacillus, and 2% Gram-negative bacillus in the nanofiber masks (Figure 14). For the control group, the Gram stain illustrates the presence of both Gram-positive and Gram-negative bacteria: Coccus, Bacillus, and spirochetes morphology. Based on the analysis, 44% were Gram-negative cocci, 9% Gram-positive cocci, 14% Gram-negative bacillus, 32% Gram-positive bacillus, and 1% spirochetes and unknown bacteria.

**Figure 15:**
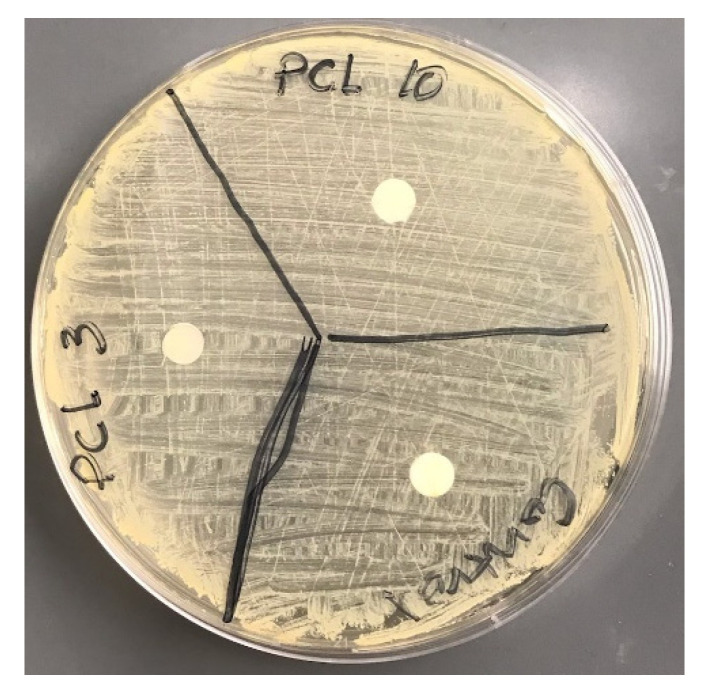
Inhibition zone experiment on a control, PCL-3, and PCL-10 disc in a TSA agar plate.

Our anti-bacterial tests using disc diffusion methods found no inhibition zone for any sample group (Figure 15). Therefore, we can conclude that none of the samples have anti-bacterial properties.

## 4. Discussion

This study found a significantly lower Young’s modulus and tensile strength for the polypropylene fabric material used as the middle filter material of generic face mask, compared to both of our tested PCL nanofiber materials. Comparing the polypropylene values with the literature, it can be seen that the material strength and flexibility of the polypropylene cloth used in our surgical mask were significantly lower compared to other scientific reports using the same polypropylene cloth (maximum tensile stress: 20–30 MPa, Young’s modulus: 800–1300 MPa) [24,25]. This difference could be due to the fabrication techniques and internal architecture of the tested materials. The tensile strength of the PCL-3 and PCL-10 cloths were found to be in agreement with the range for tensile strength (2.5–27.5 MPa) with different porosities and width to length ratios of PCL nanofiber membranes [26]. 

This study observed better hydrophobicity on the control sample than the PCL samples; however, the water absorption properties of the control samples were significantly higher compared to the PCL samples. These results can be justified by the fact that contact angle tests were conducted up to 60 s, where water absorption tests were conducted after 5 min. These results indicate that the hydrophobicity of the control material depends on the soaking time.

The DSC results suggest that the cooling rate affects the crystallization of the nanofibers when they are fabricated and deposited onto the collector. Therefore, the fiber properties induced by rapid stretching of an electrical jet and rapid evaporation of the solvent need systematic investigation through thermal analysis. The results are consistent with the recent publication by Nguyen et al. [27], where they reported that thermal treatment increases the melting temperature, degree of crystallinity, and crystallite size of PEO nanofibers. However, a detailed explanation of the thermal analysis is beyond the scope of the present paper, although we will conduct a detailed analysis on the confined crystallization in electrospun nanofibers. 

This study designed its filtration test based on the maximal human expiratory (233 ± 84 cm H_2_O) pressure [1], which is equivalent to 172 mm Hg pressure. This study conducted filtration tests at the maximum vacuum pressure (20 in Hg, which is equivalent to 508 mm of Hg pressure) to see the filtration capability and mechanical strength at this maximum vacuum pressure. A filtration efficiency test under normal breathing pressure could be conducted as a future study.

Airborne bacteria are vital biochemical components of bio-aerosols and play a critical role in ecosystems. Bacteria in high concentrations in the environment can cause biological air pollution as well as a wide range of diseases [28]. Although identifying types of bacteria (outdoor or indoor) depends on the location, temperature, and environment conditions, Gram-positive and -negative rods and unknown bacteria concentrations were significantly higher outdoors, while Gram-positive cocci concentrations were significantly higher indoors (39 vs. 24 CFU/m^3^, P 0.001). Gram-negative cocci were also more abundant outside [29]. This study has confirmed the presence of aerosol bacteria on both masks’ surfaces, with a slight difference between the type of bacteria and percentage that the masks trapped during the experimental phase. However, the lab-made nanofiber mask with PCL-10 cloth has the potential to compete with current surgical masks. The results of this study indicate that the percentage of Gram-positive cocci bacteria that the nanofiber masks trapped, as also previously proved to be more abundant in indoor environments, is significantly higher than the surgical masks (53% vs. 9%) due to the lower porosity of the PCL mask structure vs the surgical mask (control). The future goal of this research will be more focused on proving the hypothesis that the nanofiber masks are more qualified and applicable compared to the surgical masks by conducting different tests, involving more locations, and increasing the sample size.

This study is limited to the use of the middle polypropylene fabric layer of a Henry Schein Earloop surgical mask for control material. The reason for this selection of surgical mask was that it is one of the most widely used masks for general purposes. We also studied two groups of PCL samples, as the objective of the study was to measure the effects of electrospun polycaprolactone nanofiber mesh for masks with varying electrospinning time.

Pore size is one of the most important factors for the development of an antibacterial mask. This study found that PCL-10 has a suitable pore size to be qualified as a filter layer in an antibacterial mask. However, PCL itself is not antibacterial, although PCL can be bonded with an antibacterial agent to make the PCL antibacterial and allow it to be used for the development of antibacterial masks. The authors previously reported antibacterial activities of MgO nanoparticles incorporated into PCL membranes using Staphylococcus aureus [30]. Presently, the world is consumed by the pandemic and we have constantly worn masks all year; PCL with antibacterial agents (e.g., Mg, Ag, gentamicin, etc.) may aid us with the future production of face masks with antibacterial properties. A PCL nanofiber cloth has the potential to be used for such a mask to protect us against airborne microorganism particulates. 

## 5. Conclusions

This study has shown that polycaprolactone nanofiber cloth could be more effective than the generic surgical masks currently used by medical workers. The pore diameter of electrospun PCL nanofiber cloth is controllable and can be made smaller than the current pore size of the procedure masks. Our single fabricated PCL-10 nanofiber membranes show pores with an average diameter of 1.42 ± 0.34 µm. There were no visible pores when we assembled two layers of PCL nanofiber membranes. Thermal analysis showed that our PCL cloth can be used in an environment below 54 °C. Our filtration experiment found that the PCL fiber cloth could withstand a negative pressure of at least 1265 cm H_2_O without any breakage, and the mask was able to capture airborne particles. This study concluded that a minimum of two layers of PCL nanofiber membranes covered by a fabric might be capable of filtering out molecules less than 120 nm. A PCL membrane has adequate strength to withstand maximum inspiratory and expiratory pressures and can be sutured on to generic fabrics. Due to the properties mentioned above, it may also be suitable in industries that generate toxic particles that may be released into the surroundings. These industries may be able to implement filter usage to protect the environment. Therefore, PCL nanofiber cloths have multiple benefits, including being used for surgical masks to protect individuals from airborne particles containing microorganisms, and also helping prevent industries from polluting the environment.

## Figures and Tables

**Table 1:** Pore analysis on control (middle filter layer of surgical mask), PCL-3, and PCL-10 samples from a SEM image using line intercept method. The pore size is given in mean ± SD. In the table, * represents *p* < 0.05 compared to control.

Specimen Type	Pore Sizes (Micrometer)
Surgical Mask	5.71 ± 3.41
PCL-3	4.65 ± 2.09
PCL-10	1.42 ± 0.34 *

**Table 2:** Tension test results of control, PCL-3, and PCL-10 cloth. The data are presented as mean ± standard of error for sample size, *n* = 5. In the figure, * refers *p* < 0.05 compared to control.

Experimental Parameters	Control	PCL-3	PCL-10
Young’s Modulus (MPa)	15.34 ± 2.86	18.18 ± 4.41	33.96 ± 5.77 *
Tensile strength (MPa)	1.49 ± 0.29	4.96 ± 0.19 *	6.33 ± 0.78 *

## Data Availability

Data Sharing is not applicable.
